# Endoplasmic reticulum stress and eIF2α phosphorylation: The Achilles heel of pancreatic β cells

**DOI:** 10.1016/j.molmet.2017.06.001

**Published:** 2017-07-12

**Authors:** Miriam Cnop, Sanna Toivonen, Mariana Igoillo-Esteve, Paraskevi Salpea

**Affiliations:** 1 ULB Center for Diabetes Research, Faculty of Medicine, Université Libre de Bruxelles, Brussels, Belgium; 2 Division of Endocrinology, Erasmus Hospital, Université Libre de Bruxelles, Brussels, Belgium

**Keywords:** Diabetes, Endoplasmic reticulum stress, eIF2α, Pancreatic β cell, Insulin, Islet, ATF, activating transcription factor, CHOP, C/EBP homologous protein, CReP, constitutive repressor of eIF2α phosphorylation, CRISPR, clustered regularly interspaced short palindromic repeats, eIF2, eukaryotic translation initiation factor 2, ER, endoplasmic reticulum, ERAD, ER-associated degradation, GCN2, general control non-derepressible-2, GIP, glucose-dependent insulinotropic polypeptide, GLP-1, glucagon-like peptide 1, GWAS, genome-wide association study, hESC, human embryonic stem cell, hiPSC, human induced pluripotent stem cell, HNF1A, hepatocyte nuclear factor 1-α, hPSC, human pluripotent stem cell, HRI, heme-regulated inhibitor kinase, IAPP, islet amyloid polypeptide, IER3IP1, immediate early response-3 interacting protein-1, IRE1, inositol-requiring protein-1, ISR, integrated stress response, MEHMO, mental retardation, epilepsy, hypogonadism and -genitalism, microcephaly and obesity, MODY, maturity-onset diabetes of the young, NRF2, nuclear factor, erythroid 2 like 2, PBA, 4-phenyl butyric acid, Pdx1, pancreatic duodenal homeobox 1, PERK, PKR-like ER kinase, PKR, protein kinase RNA, PP1, protein phosphatase 1, PPA, phenylpropenoic acid glucoside, RIDD, regulated IRE1-dependent decay, RyR2, type 2 ryanodine receptor/Ca^2+^ release channel, SERCA, sarcoendoplasmic reticulum Ca^2+^ ATPase, TUDCA, taurine-conjugated ursodeoxycholic acid derivative, uORF, upstream open reading frame, UPR, unfolded protein response, WFS, Wolfram syndrome, XBP1, X-box binding protein 1

## Abstract

**Background:**

Pancreatic β cell dysfunction and death are central in the pathogenesis of most if not all forms of diabetes. Understanding the molecular mechanisms underlying β cell failure is important to develop β cell protective approaches.

**Scope of review:**

Here we review the role of endoplasmic reticulum stress and dysregulated endoplasmic reticulum stress signaling in β cell failure in monogenic and polygenic forms of diabetes. There is substantial evidence for the presence of endoplasmic reticulum stress in β cells in type 1 and type 2 diabetes. Direct evidence for the importance of this stress response is provided by an increasing number of monogenic forms of diabetes. In particular, mutations in the PERK branch of the unfolded protein response provide insight into its importance for human β cell function and survival. The knowledge gained from different rodent models is reviewed. More disease- and patient-relevant models, using human induced pluripotent stem cells differentiated into β cells, will further advance our understanding of pathogenic mechanisms. Finally, we review the therapeutic modulation of endoplasmic reticulum stress and signaling in β cells.

**Major conclusions:**

Pancreatic β cells are sensitive to excessive endoplasmic reticulum stress and dysregulated eIF2α phosphorylation, as indicated by transcriptome data, monogenic forms of diabetes and pharmacological studies. This should be taken into consideration when devising new therapeutic approaches for diabetes.

## 1 Introduction

The prevalence of diabetes is reaching epidemic proportions, with an estimated 420 million people affected globally [1]. Most patients have polygenic forms of diabetes. 10–15% have type 1 diabetes, where the immune system selectively targets insulin-producing pancreatic β cells. 80% have type 2 diabetes, an ill-defined condition of pancreatic β cell dysfunction in a context of insulin resistance. An increasing number of monogenic forms of diabetes is being discovered [2], [3], in which a mutation in a single gene causes disease. Monogenic forms of diabetes are uncommon to very rare, but from a pathophysiological perspective they provide important insight into molecules and pathways that are crucial to develop and maintain human β cell function. Variants in monogenic diabetes genes have been shown contribute to type 2 diabetes risk [3]. These monogenic forms of diabetes can be used as experiments of nature that provide us with human knockout models for key aspects of islet biology. From a clinical perspective, the diagnosis of monogenic forms of diabetes is important because for some of these forms of diabetes therapy can be tailored to the specific genetic defect [4]. Here we will discuss the compelling evidence for the role of endoplasmic reticulum (ER) stress and dysregulated ER stress signaling in β cell failure and human diabetes. We will review the rodent models classically used to study disease mechanisms, novel human induced pluripotent stem cell (hiPSC)-based models, and therapeutic modulation of ER stress and signaling in β cells.

## 2 Endoplasmic reticulum stress

**Figure 1:**
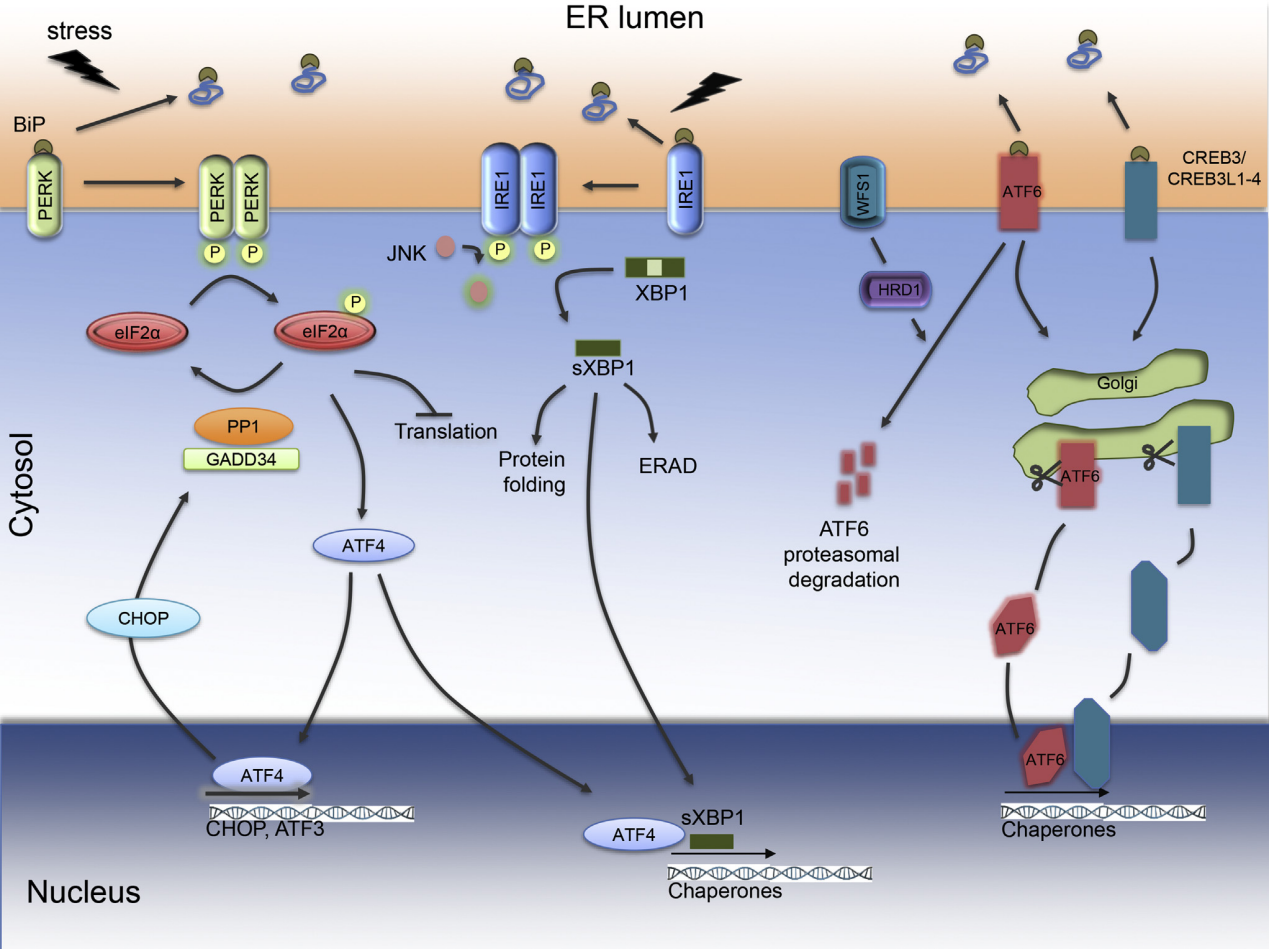
Endoplasmic reticulum stress signaling. ER stress leads to increased binding of the ER chaperone BiP to misfolded proteins in the ER lumen, causing the dissociation of BiP from the ER stress transducers PERK, IRE1, and ATF6, resulting in their activation. Activated (phosphorylated) PERK phosphorylates eIF2α and thereby attenuates general protein translation to relieve the ER workload during stress. In parallel, eIF2α phosphorylation enhances ATF4 translation. ATF4 induces transcription of chaperones and CHOP. CHOP induces expression of GADD34, which targets PP1 to eIF2α for dephosphorylation and relief of translational inhibition. IRE1 activation (phosphorylation) causes the splicing of XBP1 mRNA, generating the transcription factor sXBP1. sXBP1 upregulates the expression of chaperones, folding enzymes and components of the ERAD machinery. Activated ATF6 translocates to the Golgi where it is cleaved to a mature transcription factor that will drive chaperone expression. WFS1 inhibits ATF6 through ubiquitination and proteasomal degradation, by targeting HRD1 (E3 ubiquitin ligase) to ATF6. CREB3 and CREB3L1 through CREB3L4 are cell type- and context-specific ER stress transducers that are cleaved/activated in a similar manner as ATF6.

ER stress is defined as an imbalance between the protein folding capacity of the organelle and the functional demand that is placed on it. Such an imbalance leads to accumulation of unfolded or misfolded proteins in the ER lumen. In order to restore ER homeostasis, cells trigger the ER stress response, also known as the unfolded protein response (UPR) [5]. This adaptive response aims to increase the functional capacity of the organelle and to decrease the arrival of newly synthesized proteins. The former is achieved by transcriptional upregulation of folding enzymes and chaperones and expansion of ER size. The latter is done by attenuating protein translation through phosphorylation of the eukaryotic translation initiation factor 2 (eIF2) and mRNA degradation. ER stress transducers present in the ER membrane orchestrate this response; their luminal domain senses the unfolded protein stress and their cytoplasmic domain signals to cytosol and nucleus. Two fundamental branches of the UPR are under the control of the canonical ER stress transducers protein kinase RNA (PKR)-like ER kinase (PERK) and inositol-requiring protein-1 (IRE1) (Figure 1). A third branch of the UPR is activated by activating transcription factor 6 (ATF6). In addition to these classical ER stress transducers, the CREB3 and CREB3L1-4 transcription factors elicit UPR signaling in a cell type- and context-specific manner [6].

**Figure 2:**
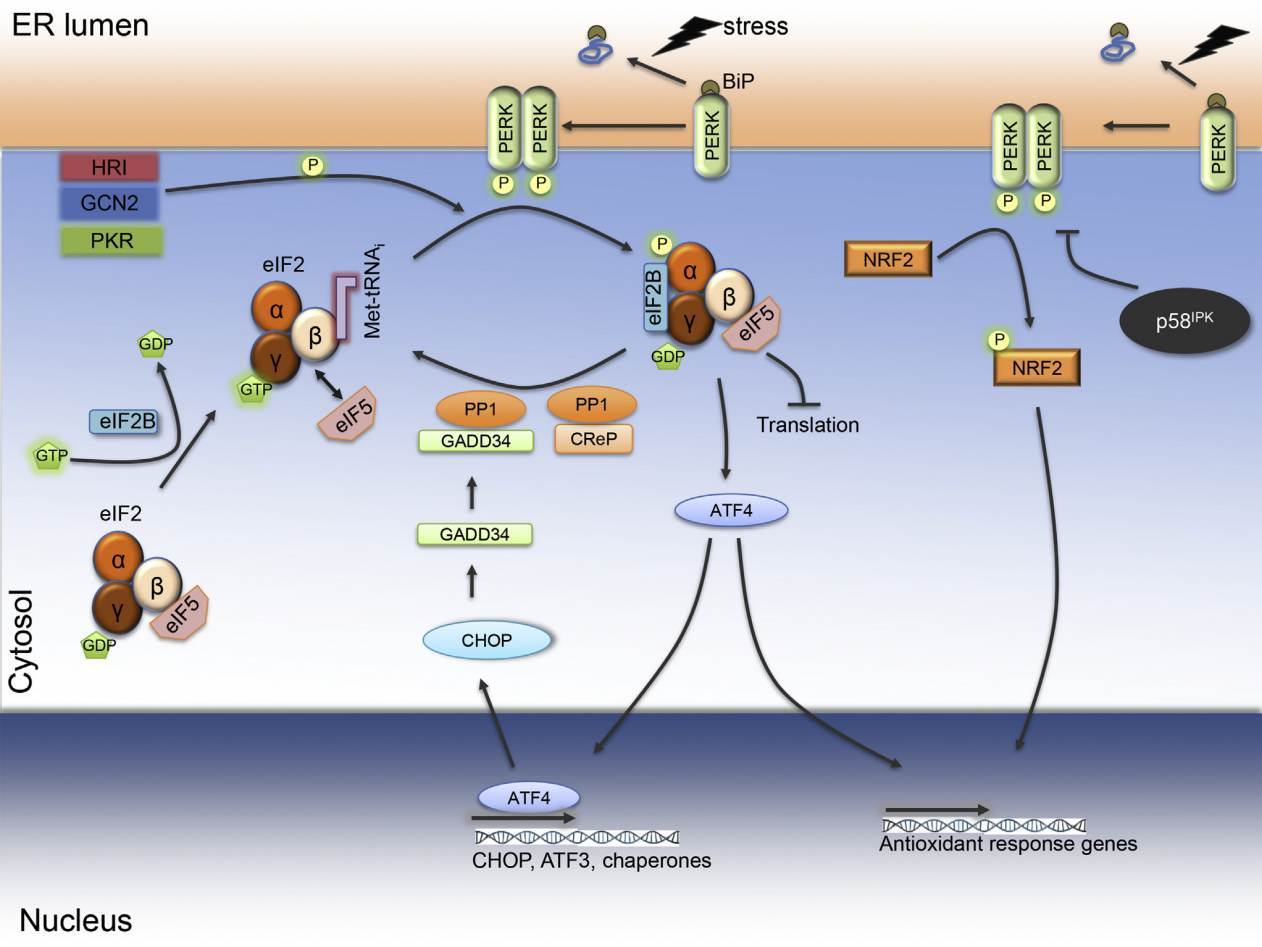
Regulation of eIF2α phosphorylation. ER stress leads to PERK phosphorylation and activation, and phosphorylation of eIF2α and NRF2, an antioxidant response transcription factor. The eIF2 protein consists of three subunits, eIF2α, -β, and -γ. Active eIF2 has a non-phosphorylated eIF2α and low affinity to the guanine nucleotide exchange factor eIF2B. In that state eIF2B exchanges GDP to GTP from the eIF2γ subunit, ensuring its active state. Non-phosphorylated eIF2α, eIF2β, GTP-loaded eIF2γ, methionyl tRNA (Met-tRNA_i_), and eIF5 form the ternary complex. Upon start site recognition eIF2 and eIF5 dissociate from the complex, and translation initiation and elongation ensue. eIF2α phosphorylation at Ser51 increases its affinity for eIF2B and eIF5, reduces the guanine nucleotide exchange that slows down the formation of the ternary complex, and thereby attenuates translation initiation. In parallel, this initiates ATF4 translation and downstream expression of chaperones, antioxidant response genes, CHOP and GADD34. GADD34-bound PP1 dephosphorylates eIF2α and ends translational inhibition and ATF4 expression/signaling. CReP is another constitutively expressed non-enzymatic cofactor for PP1 that tonically keeps eIF2α phosphorylation down. The BiP co-chaperone p58^IPK^ inhibits PERK and downstream signaling. eIF2α can also be phosphorylated by the non-ER stress-related kinases HRI, PKR, and GCN2.

PERK is the eIF2 kinase activated by ER stress. Three other eIF2 kinases are activated in conditions of heme deficiency (heme-regulated inhibitor kinase, HRI), double-stranded RNA produced during viral infection (PKR), and amino acid starvation and tRNA uncharging (general control non-derepressible-2, GCN2) (Figure 2). These four kinases phosphorylate the α subunit of eIF2 in position Ser51 and thereby attenuate translation initiation, the rate-limiting step of protein synthesis [7]. The α, β, and γ subunits of eIF2 form the ternary complex together with GTP and initiator methionyl tRNA (Met-tRNA_i_) (Figure 2). The ternary complex, together with other initiation factors and the 40S ribosome, binds an mRNA and scans it to identify the AUG codon translation start site [8]. Start site recognition leads to hydrolysis by eIF5 of eIF2γ-bound GTP. eIF2 and eIF5 are then released from the 40S ribosome, which allows the 60S ribosome to bind. Protein elongation ensues. To engage in new rounds of translation initiation, the GDP on released eIF2γ needs to be exchanged for GTP in a reaction catalyzed by the guanine nucleotide exchange factor eIF2B. eIF2α phosphorylation inhibits eIF2B and thereby prevents reloading of GTP into the ternary complex (Figure 2). eIF5 further inhibits GDP dissociation from eIF2γ when eIF2α is phosphorylated [9]. The abundance of expression of eIF2α, eIF5 and the five subunits of eIF2B may regulate the extent to which eIF2α phosphorylation inhibits translation initiation [10]. eIF2α phosphorylation paradoxically increases translation of certain mRNAs with short inhibitory upstream open reading frames (uORFs) in their 5′ untranslated region, such as ATF4 and C/EBP homologous protein (CHOP). eIF2α phosphorylation and reduced ternary complex formation lead to ribosomal skipping of the repressing uORF and initiation of ATF4 translation at the start codon. In the case of CHOP, a single inhibitory uORF with a poor translation initiation context (i.e. a poor Kozak consensus sequence) is skipped [11]. Translation can be sustained via other mechanisms. In pancreatic β cells, translation of pancreatic duodenal homeobox 1 (Pdx1) is maintained during eΙF2α phosphorylation by an internal ribosome entry site in its 5′ untranslated region that enhances cap-independent Pdx1 translation [12]. ATF4 transcriptionally induces genes involved in amino acid transport and metabolism, glutathione biosynthesis, and an antioxidant response [13]. ATF4 induces ATF3 and CHOP, two transcription factors that further modify gene expression during ER stress. Given that signaling downstream of phospho-eIF2α can be elicited by four eIF2 kinases activated by different stresses, this branch has been called the integrated stress response (ISR) [13]. PERK also phosphorylates nuclear factor, erythroid 2 like 2 (NRF2), and thereby promotes antioxidant gene expression.

The PERK pathway is subject to negative feedback inhibition to terminate translational repression through a number of mechanisms (Figure 2). During ER stress, growth arrest and DNA damage inducible gene 34 (GADD34) is induced and will target protein phosphatase 1 (PP1) to dephosphorylate eIF2α. The constitutive repressor of eIF2α phosphorylation (CReP) is expressed constitutively [14] and is further induced during ER stress [15]. Similar to GADD34, CReP serves as a non-enzymatic co-factor providing PP1 specificity for phospho-eIF2α. G-actin was shown to integrate and provide further specificity to the holophosphatase complex [16], [17]. The ER co-chaperone p58^IPK^ inhibits PERK activity [18], and also PKR [19]. These different factors attenuate or terminate eIF2α phosphorylation and downstream signaling and restore protein synthesis.

IRE1 activation leads to the splicing of the mRNA for X-box binding protein 1 (XBP1), which can then be translated into sXBP1 (Figure 1). This transcription factor upregulates genes encoding folding enzymes and chaperones, components of the ER-associated degradation (ERAD) machinery to clear terminally misfolded proteins, and a lipogenic program to expand the ER compartment. IRE1 also activates the regulated IRE1-dependent decay (RIDD) of ER-associated mRNAs, which helps to decrease the newly synthesized protein load in the ER [20], [21].

In conditions of ER stress, ATF6 translocates from the ER to the Golgi where it is cleaved by site 1 and site 2 proteases. Cleaved ATF6 transcriptionally upregulates ER chaperones such as BiP to enhance the ER folding capacity, and genes involved in lipid biosynthesis and ERAD (Figure 1).

## 3 Endoplasmic reticulum stress in polygenic diabetes

There is substantial evidence for the presence of ER stress in β cells in type 1 and type 2 diabetes [22], [23]. In pancreatic sections from type 1 diabetic patients, islet ATF3 [24] and CHOP expression were increased, but sXBP1 was comparable to controls [25]. In insulitis-positive islets, BiP expression was also increased [25]. In another study, islet ATF6 and sXBP1 levels were decreased in type 1 diabetes, especially in patients with long-standing disease [26]. This was suggested to point to defective UPR and failure to resolve ER stress.

In type 2 diabetes, islets have increased levels of p58^IPK^, CHOP, ATF3, and BiP proteins [24], [27], [28] and an enlarged ER, an ultrastructural hallmark of the UPR [29]. Engin et al. observed variable but overall decreased ATF6 and sXBP1 expression in islets of type 2 diabetic patients and suggested that, in the long term, a deficient β cell UPR leads to β cell demise [30]. Interestingly, little if any eIF2α phosphorylation was detected in β cells; it was present in islet non-β cells and decreased in type 2 diabetes [30].

The apparent discordance between directional changes in expression of ER stress markers in islets from diabetic patients may be because post-mortem pancreas allows assessment at one given time point only; the kinetics of ER stress signaling may differ for different UPR proteins. Compared to inbred laboratory animal studies, human tissue will be much more variable and influenced by patient characteristics, concurrent disease, differences in tissue procurement, etc.

The complexity of these polygenic diseases and multifactorial etiology means that a variety of triggers can cause ER stress in type 1 and type 2 diabetes. Among these are genetic variants (see below) and exposure to cytokines and viral infections in type 1 diabetes and to free fatty acids, glucose, islet amyloid polypeptide (IAPP), and increased β cell workload due to insulin resistance in type 2 diabetes. The reader is referred to comprehensive reviews on the topic [22], [23], [31].

There is evidence for therapeutic modulation of ER stress and improved β cell outcomes in experimental models (see below). Little pharmacological evidence is available in man, however. This is due to the pleiotropic effects of some of the drugs used, such as glucagon-like peptide 1 (GLP-1) analogs, on the one hand, and the difficulty to assess β cell ER stress in man, on the other. Increased circulating proinsulin/insulin or proinsulin/C-peptide ratios have been used as an indicator of β cell ER stress in individuals progressing to type 1 diabetes [32]. This biomarker, however, is not entirely specific for ER stress. The discovery of a method to detect β cell ER stress *in vivo* by circulating biomarker or imaging tools, while challenging, would be an important development.

## 4 Endoplasmic reticulum stress in monogenic diabetes

In contrast to the complexity of polygenic forms of diabetes in which environmental factors play important roles, monogenic forms of diabetes provide unmistakable evidence for the crucial role of a molecule in a particular process in man. To quote Yossi Schlessinger in reference [33]: “Genetics doesn't lie. It doesn't tell you the mechanism, but it doesn't lie”.

### 4.1 Akita insulin: the prototype of ER stress-related diabetes

The proteins synthesized in the ER comprise all secreted and membrane expressed proteins. Any mutation leading to misfolding of these proteins in the ER can theoretically cause ER stress and β cell demise and diabetes. The Akita insulin mutation, initially described in mice [34] (see below) and then as a cause of neonatal diabetes in man [35], can be seen as the prototype of ER stress-related diabetes. This dominant C96Y *INS* mutation causes ER stress by the creation of proinsulin that misfolds because the B7-A7 disulfide bridge cannot be formed. In spite of 50% of synthesized insulin being normal in humans (and 75% in heterozygous *Ins2*^Akita/+^ mice, given that rodents have two insulin genes *Ins1* and *Ins2*), the proteotoxicity is such that insulin secretion is impaired *in utero* [35] or rapidly after birth [36], causing neonatal diabetes. Neonatal diabetes is typically defined as diabetes with onset before the age of 6 months, but some *INS* mutation patients develop diabetes later in childhood or young adulthood. Patients with dominant *INS* mutations have severe hyperglycemia at diagnosis, and often present with ketoacidosis, indicating marked insulin deficiency [35], [36]. Initially they may have detectable or even elevated circulating C-peptide levels, pointing to the presence of residual β cell mass and function, but this falls rapidly and often becomes undetectable [37]. A mutation in the neighboring cysteine C95 causes a similar phenotype in the Munich mouse [38] and in man [36] by impairing the formation of the intra-A chain A6-A11 disulfide bond. Most dominant *INS* mutations have been shown or are predicted to lead to proinsulin misfolding [39]. This causes ER stress that – in spite of attempts by the UPR – cannot be resolved, and triggers β cell apoptosis at least in part via CHOP [40]. No postmortem studies are available of mutant *INS* patients' pancreas, but the mouse models show marked reductions in β cell mass. Prior to β cell depletion, other mechanisms may also contribute, including impaired production of wild type insulin. This may be due to perturbations in the ER chaperone, protein folding and oxidizing potential due to chronic ER stress, or due to interactions between wild type and mutant proinsulin molecules. *INS* mutations that result in removal of a native cysteine or aberrant introduction of a new one cause unpaired cysteines to be available for intermolecular disulfide bond formation. This leads to impaired intramolecular disulfide bond generation in wild type proinsulin, and this misfolded proinsulin is then targeted for ERAD [41].

### 4.2 Diabetes caused by dysregulated endoplasmic reticulum stress signaling

#### 4.2.1 The PERK branch

##### 4.2.1.1 EIF2AK3 diabetes in Wolcott-Rallison syndrome

**Figure 3:**
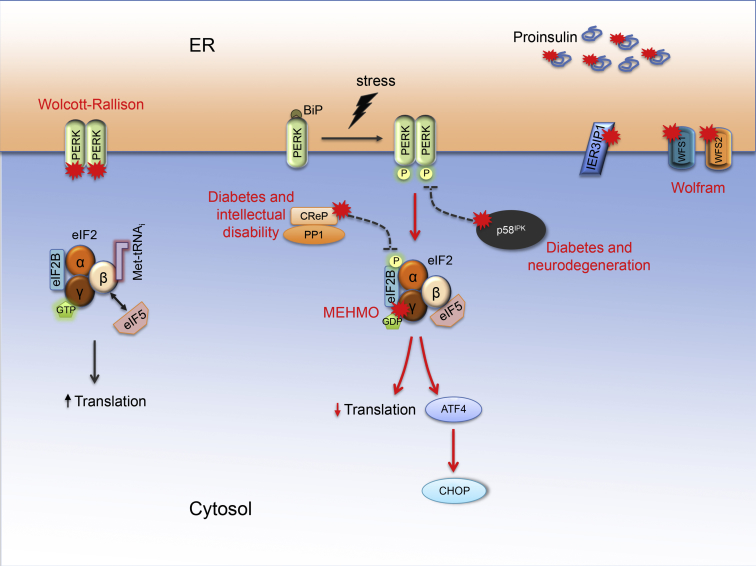
Monogenic diabetes due to excessive or dysregulated endoplasmic reticulum stress signaling. Four monogenic forms of diabetes pertain to the PERK branch of the UPR. Inactivating mutations in *EIF2AK3*, encoding PERK, cause Wolcott-Rallison syndrome (left). In these patients, PERK is unable to phosphorylate eIF2α, leading to absent PERK signaling, loss of translational control, ER stress and β cell loss. In the three other monogenic forms (middle), eIF2α phosphorylation/inactivation and downstream signaling are enhanced. Loss-of-function mutations in *DNAJC3*, encoding p58^IPK^, cause diabetes and neurodegenerative features. The p58^IPK^ inactivation results in increased PERK activity and eIF2α phosphorylation. Loss-of-function mutations in *PPP1R15B*, encoding CReP, causes a syndrome comprising diabetes, short stature, intellectual disability, and microcephaly. The *PPP1R15B* mutation destabilizes the CReP-PP1 holophosphatase complex and thereby enhances eIF2α phosphorylation. Mutations in *EIF2S3*, encoding eIF2γ, cause MEHMO syndrome (mental retardation, epilepsy, hypogonadism and hypogenitalism, microcephaly, and obesity). These damaging *EIF2S3* mutations impair eIF2 function and enhance downstream signaling. Missense mutations in *IER3IP1* lead to a Wolcott-Rallison-like syndrome of microcephaly, epilepsy, and neonatal diabetes. Recessive mutations in *WFS1* and *WFS2* lead to Wolfram syndrome. *INS* mutations that impair proinsulin folding cause β cell demise and neonatal diabetes.

Recessive mutations in *EIF2AK3*, encoding PERK, give rise to Wolcott-Rallison syndrome [42] (Figure 3). The disease is characterized by neonatal or early infancy-onset non-autoimmune, insulin-dependent diabetes, often with a ketoacidotic presentation. Associated features are growth retardation, epiphyseal dysplasia, hepatic steatosis and dysfunction, exocrine pancreas insufficiency, intellectual disability, and microcephaly [43]. The mutations are frameshift, nonsense, or missense mutations in the kinase domains of PERK [43], [44]. The severity of the loss of PERK kinase function may be correlated to age at diabetes onset [44]. Loss of PERK function leads to β cell dysfunction and death. Post-mortem pancreata from two Wolcott-Rallison syndrome patients showed a very marked reduction of β cells in the islets, while α, δ, and pp cells were normal [45]. Heterozygous carriers of *EIF2AK3* mutations do not present with diabetes [44]. Variants in *EIF2AK3* have been associated with increased risk for type 1 and type 2 diabetes [46], [47], [48]. Wolcott-Rallison syndrome shows that absence of PERK function and inability to phosphorylate eIF2α in conditions of ER stress leads to β cell demise. Interestingly, the following three forms of diabetes demonstrate that β cells neither tolerate the reverse, i.e. excessive eIF2α phosphorylation/inactivation.

##### 4.2.1.2 EIF2S3 diabetes

Mutations in *EIF2S1*, which encodes eIF2α, have not been described in man. Missense mutations in eIF2γ (encoded by *EIF2S3*) that disrupt eIF2 complex integrity have been identified in patients with X-linked microcephaly, epilepsy, and micropenis [49]. In two additional families, neonatal or young-onset hypoglycemia was reported [50]. A recent study identified *EIF2S3* mutations in patients with MEHMO syndrome (mental retardation, epilepsy, hypogonadism and -genitalism, microcephaly, and obesity) [51]. All three patients with this damaging *EIF2S3* frameshift mutation who were alive by 1 year developed non-autoimmune insulin-dependent diabetes before that age; in one the presentation was by ketoacidosis. In elegant functional studies, Skopkova et al. showed that the frameshift mutation impairs eIF2 function, reducing fidelity of translation start site selection and increasing ATF4 translation and CHOP expression in the ISR [51] (Figure 3).

It should be noted that mutations in any of the five *EIF2B* genes that encode the eIF2B subunits are causative of vanishing white matter leukodystrophy, but not diabetes [52]. Reduced activity of eIF2B, the guanine nucleotide exchange factor that converts inactive eIF2-GDP into active eIF2-GTP, enhances signaling in the ISR [53], but does not phenocopy the *EIF2S3* mutations.

##### 4.2.1.3 DNAJC3 diabetes

Synofzik et al. described recessive *DNAJC3* loss-of-function mutations in two families with diabetes and neurodegenerative features, including ataxia, upper-motor-neuron damage, peripheral neuropathy, hearing loss, and cerebral atrophy [54]. *DNAJC3* encodes p58^IPK^, a BiP co-chaperone that inhibits PERK activity and downstream signaling (Figure 3). Diabetes developed between 11 and 18 years, was non-autoimmune and insulin requiring even if some residual endogenous insulin secretion was present [54]. Findings 10 years earlier of non-ketotic diabetes and β cell apoptosis in a p58^IPK^ knockout mouse are consistent with the patients' phenotype [55].

##### 4.2.1.4 PPP1R15B diabetes

We reported a homozygous *PPP1R15B* mutation in two siblings with diabetes, short stature, intellectual disability, and microcephaly [15]. The index case was diagnosed with non-autoimmune diabetes at age 15 years; he remained C-peptide positive over the years. His sister presented with ketotic diabetes at age 28 years. *PPP1R15B* encodes CReP, a non-enzymatic cofactor for PP1 that dephosphorylates eIF2α. The R658C missense mutation affects a conserved amino acid in the PP1 binding domain that leads to destabilization of the CReP-PP1 holophosphatase complex and diminished eIF2α dephosphorylation [15]. CReP-deficient β cells had reduced insulin content and secretion, and were more sensitive to apoptosis [15]. Additional *PPP1R15B* patients with intellectual disability, microcephaly, and short stature have since been identified [56], [57]. At age 6 months, the siblings reported by Mohammad et al. underwent liver transplantation for cirrhosis [57]. Diabetes was not reported in these patients but their age at the time of publication was young (below 6 years).

#### 4.2.2 IER3IP1 diabetes

Missense mutations in *IER3IP1* (immediate early response-3 interacting protein-1) lead to a Wolcott-Rallison-like syndrome characterized by microcephaly with simplified gyration, epilepsy, and neonatal diabetes [58], [59], [60]. *IER3IP1* codes for a small (10 kDa) highly conserved ER protein [61] that is highly expressed in the developing brain and pancreas [60]. Postmortem analysis of brain and pancreas of IER3IP1-deficient patients showed increased neuronal apoptosis and reduced β cell mass [60]. IER3IP1 may regulate cell differentiation and death; it has not been reported whether its deficiency alters ER homeostasis or the UPR.

#### 4.2.3 The ATF6 and IRE1 branches

In contrast to the multiple loss-of-function mutations in genes involved in PERK signaling identified in diabetic patients (Figure 3), there is no human monogenic evidence for a vital role of the ATF6 and IRE1 branches in β cells. Polymorphisms in *ATF6* have been associated with increased type 2 diabetes risk in Dutch Caucasians and Pima Indians [62], [63], but this was not confirmed in other reports in a variety of populations [64], [65]. An association has also been reported between an *XBP1* variant and type 2 diabetes in Han Chinese [66].

#### 4.2.4 WFS1 and WFS2 diabetes in Wolfram syndrome

Recessive mutations in *WFS1* and *WFS2* lead to Wolfram syndrome 1 and 2, respectively, two autosomal recessive disorders with young-onset diabetes, optic nerve atrophy, and deafness [67]. Other manifestations include ataxia, dysphagia, nystagmus, apnea related to brain stem atrophy [68], psychiatric manifestations, and seizures [68], [69]. The prognosis of this orphan disease is poor. Most Wolfram patients carry mutations in *WFS1* [70]; more than 100 different mutations have been identified, including missense, nonsense, and insertion/deletion aberrations, most often in exon 8 [70], [71], [72], [73], [74], [75]. *WFS1* encodes for wolframin, a 100 kDa transmembrane glycoprotein localized in the ER [68]. Wolframin is highly expressed in pancreatic β cells, with low or no expression in α cells or exocrine pancreas [76], [77]. Wolframin mRNA and protein expression are upregulated by ER stress [76]. In β cells wolframin controls the steady state levels and activity of ATF6 [78]. Under non-stressed conditions, wolframin recruits ATF6 to the E3 ligase Hrd1 for ATF6 ubiquitination and proteasomal degradation (Figure 1). In stress conditions, ATF6 dissociates from wolframin, is activated, and regulates gene expression. Wolframin deficiency leads to ATF6 hyperactivation and ER stress-mediated β cell dysfunction and apoptosis [77], [78], [79]. This is in keeping with the reduced β cell mass observed in postmortem pancreata from Wolfram syndrome patients [80]. Wolframin deletion in β cells induces CHOP, ATF4, BiP, and sXBP1 expression and reduces insulin synthesis [76]. Wolframin regulates ER Ca^2+^ homeostasis by modulating the activity and turnover of the sarcoendoplasmic reticulum Ca^2+^ ATPase (SERCA) [81], [82]. Wolframin-deficient β cells and neurons have reduced ER Ca^2+^ [81] and increased cytosolic Ca^2+^ levels, leading to activation of the Ca^2+^-dependent cysteine protease calpain and cell death [83]. The exact function of wolframin and how this protein modulates ER Ca^2+^ remain to be elucidated.

Common polymorphisms in *WFS1* have been linked to susceptibility for type 2 diabetes [84], [85], [86], [87]. Some of the variants are intronic with no evident impact on WFS1 mRNA expression or biological function. *WFS1* variants have also been associated with type 1 diabetes in Japanese [88].

Wolfram syndrome 2 is caused by mutations in *WFS2* (also called *CISD2*), which encodes for Miner1 (also named ERIS) [89], [90], an iron-sulfur cluster-containing protein localized in mitochondria-associated ER membranes and mitochondria [91], [92]. The function of Miner1 is unknown. It has been proposed that, through its iron-sulfur cluster-mediated redox capability, Miner1 contributes to ER protein modifications and ER stress response [91]. It may also interact with mitoNEET, another iron-sulfur cluster-containing protein localized in the outer mitochondrial membrane. Both proteins would respond to similar redox stimuli and communicate via electron transfer, contributing to ER-mitochondrial crosstalk, which is important for Ca^2+^ transfer between organelles and cell function [91]. As for wolframin, Miner1 deficiency leads to calpain 2-dependent β cell apoptosis, but in a Ca^2+^-independent manner [83].

### 4.3 Maturity-onset diabetes of the young

Several monogenic forms of diabetes, including autosomal dominant maturity-onset diabetes of the young (MODY), are possibly associated with a dysregulated ER stress response. The transcription factor hepatocyte nuclear factor 1-α (HNF1α) regulates XBP1 and BiP expression; inhibition of HNF1α sensitizes β cells to ER stress-induced apoptosis [93]. HNF4α controls Anks4b expression, a BiP-interacting protein that sensitizes β cells to ER stress [94]. Pdx1 controls the expression of genes involved in disulfide bond formation, ER chaperones such as BiP, and other UPR genes including ATF4 and WFS1, and thereby increases β cell resistance to ER stress [95]. GATA4 and GATA6 maintain β cell survival by promoting ER integrity; deletion of either transcription factor induces islet BiP, sXBP1, and ATF4 expression and causes β cell ER distention [96]. GATA6 mutations cause diabetes with an age at onset that varies widely (from birth to adulthood) [97]. PAX4 also helps preserve ER integrity [98]. By regulating expression of ER and UPR genes, these transcription factors promote β cell ER function and differentiation. Polymorphisms in several MODY genes, including *HNF1A*, *HNF4A*, *HNF1B*, and *PAX4* contribute to type 2 diabetes risk [99].

## 5 Exquisite sensitivity of pancreatic β cells to dysregulated ER stress

**Figure 4:**
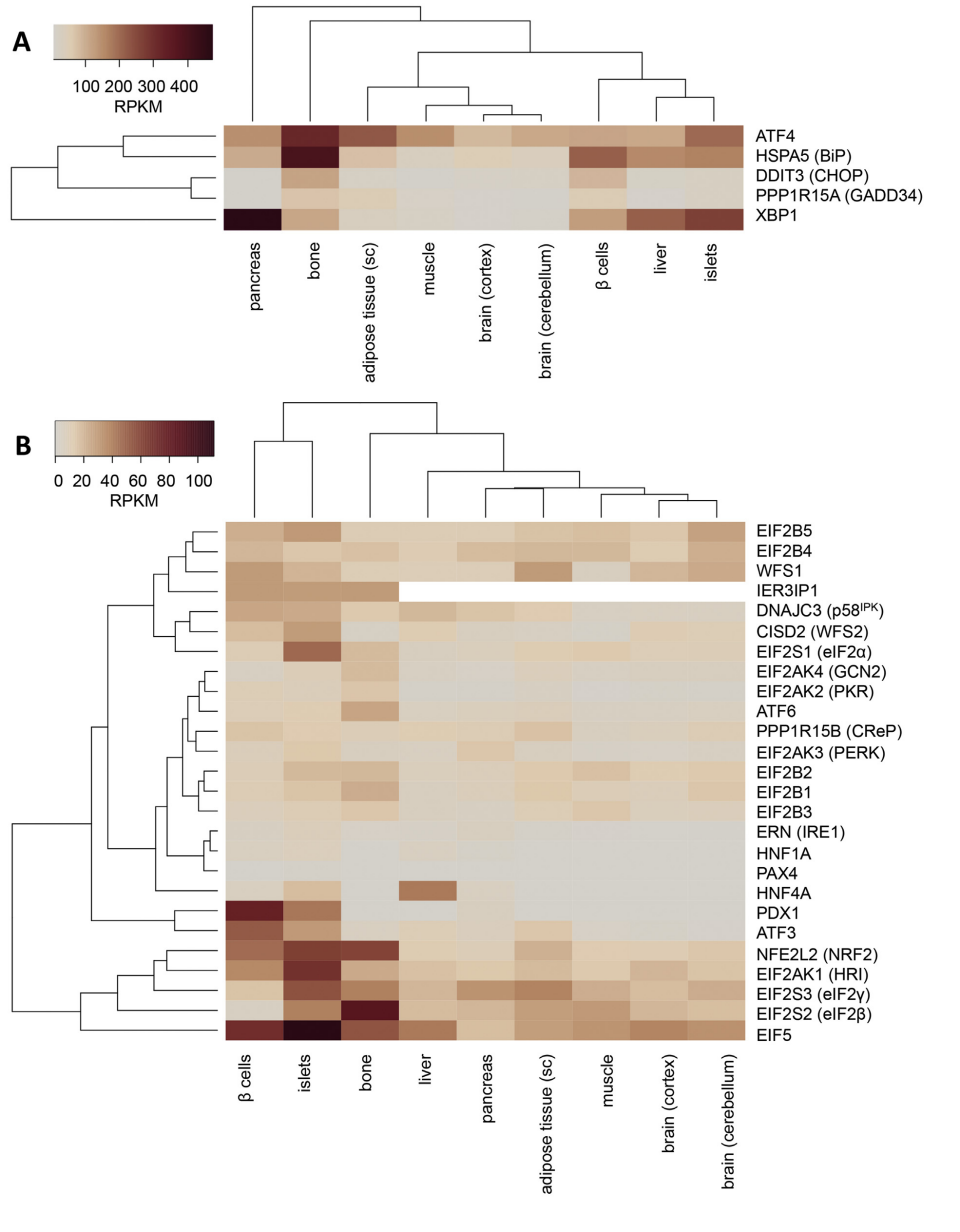
Heatmap of genes related to the endoplasmic reticulum stress response. RNA-seq gene expression levels (in RPKM) from human tissues were obtained from GTEx (v4.p1) [189]. RNA-seq data of FACS-purified human β cells were from Nica et al. [190] and human islet RNA-seq data from Eizirik et al. [191] and Cnop et al. [192]. Genes and tissue/cell types are arranged according to cluster analysis. Protein names are indicated in parenthesis when different from gene names. Panel A shows more abundantly expressed genes (100–400 RPKM) and panel B less abundant genes (0–100 RPKM). Some MODY genes are shown for comparison.

The above described monogenic forms of diabetes showcase the sensitivity of pancreatic β cells to dysregulation of the UPR. More evidence for the crucial role of the ER stress response comes from gene expression studies. Compared to a wide range of tissues, human β cells and islets abundantly express PERK and HRI, the three subunits of eIF2, eIF5, ATF4, ATF3, CHOP, GADD34, and CReP, NRF2, p58^IPK^, IRE1, XBP1, and BiP (Figure 4). WFS1 and WFS2 are also abundant in β cells and islets. Expression levels in the (exocrine) pancreas tend to be lower, except for XBP1. This gene expression pattern suggests β cells are very well equipped to signal ER stress.

## 6 Animal models to study ER stress-related diabetes

Animal models have provided insight into ER stress and the UPR and their role in β cells and glucose homeostasis. Yoshioka et al. established a mouse model of non-obese diabetes that presents severe β cell dysfunction [100], [101]. These Akita mice develop diabetes by 5–8 weeks of age, due to the C96Y missense mutation in *Ins2* that impairs formation of the B7-A7 disulfide bond [34]. This misfolded proinsulin is not transported from the ER to the Golgi, accumulates in the ER, forms complexes with the chaperone BiP, and is degraded intracellularly by ERAD. The misfolding of proinsulin leads to CHOP induction and β cell apoptosis [40]. Deletion of the *Chop* gene in the Akita model increases the functional capacity of the ER to produce folded proinsulin and limits oxidative stress, thereby delaying diabetes onset [40], [102]. Similarly, heterozygous PERK deletion delays diabetes in Akita mice [103]. In addition to the heterozygous Akita mice, the homozygous mutant *Ins2* mouse has been developed. It presents a more severe phenotype, developing diabetes by 2 weeks of age, with islet hypoplasia and few β cells [100]. In the Munich mouse, the C95S missense mutation in *Ins2* prevents formation of the A6-A11 intra-A chain disulfide bond. Heterozygous mice present severe insulinopenic diabetes by 1 month of age, while the homozygous mice develop diabetes at 18 days [38]. Both Akita and Munich mice present sex differences. β cell mass is not reduced in females, and they have a more stable and mild diabetic phenotype compared to the rapid deterioration of β cell mass and glucose homeostasis in males [34], [38]. This sex difference is not seen in human patients.

Mouse models of Wolcott-Rallison syndrome have helped to understand the importance of the PERK-eIF2α branch. The Perk^−/−^ mouse is characterized by early development of diabetes due to severe β cell loss and neuronal defects. In keeping with the phenotype of patients, murine PERK deficiency also leads to pancreatic acinar cell loss and exocrine pancreas insufficiency [45], [104], [105] and impaired osteoblast growth and function [106]. Heterozygous Perk^+/−^ mice present a mild defect in glycemic control [105], [107]. In the Perk^−/−^ model, decreased eIF2α phosphorylation leads to derepressed translational rates, ER stress, and β cell loss [105], [107]. However, other studies using tissue- and cell-specific PERK knockout mice or β cell lines showed no evidence of uncontrolled protein synthesis, activation of the UPR and apoptosis. Instead, ER-to-Golgi trafficking and ERAD were impaired, and it was suggested that ER dysfunction leads to abnormal insulin trafficking and secretion and decreased β cell proliferation [103], [108], [109], [110].

Knockout models of the other eIF2α kinases, HRI [111], GCN2 [112], and PKR [113], [114] do not present obvious defects in glucose homeostasis. A mouse model homozygous for a missense mutation (S51A) of the *eif2s1* gene completely inhibits eIF2α phosphorylation. These mice have reduced β cell mass at birth but they die within hours due to prolonged hypoglycemia as a result of reduced gluconeogenesis and liver glycogen stores [115]. Heterozygous S51A eIF2α mice develop glucose intolerance on high-fat diet. The partial dysregulation of eIF2α phosphorylation combined with metabolic stress results in a distended β cell ER, delayed proinsulin processing and decreased islet insulin content and nutrient-stimulated insulin secretion [116].

p58^IPK^ exerts one of the negative feedbacks on PERK-eIF2α signaling by inhibiting PERK activity [18], [117]. Ladiges et al. created a p58^IPK^-null mouse that has gradual β cell death and abnormal glucose homeostasis [55]. The β cells of these mice have increased caspase activity and apoptosis. This phenotype is less severe than that of the eIF2α S51A mutated mouse [115] or the PERK null mouse [105], probably because p58^IPK^ is regulatory rather than directly involved in translational attenuation. PERK-eIF2α mutants have defects in other tissues as well, while the p58^IPK^ knockout has a β cell-specific phenotype. p58^IPK+/−^ mice have no obvious phenotype [55].

In the first animal model of Wolfram syndrome, the *wfs1* gene was disrupted by exon 2 deletion [77]. These mice develop glucose intolerance or overt diabetes due to insulin deficiency as a result of impaired stimulus-secretion coupling, increased β cell sensitivity to apoptosis, and progressive β cell loss. Another study produced *wfs1* null mice by deleting exon 8 [118]. This mouse had a phenotype of glucose intolerance and growth retardation. Riggs et al. created a conditional mouse with a β cell-specific *wfs1* exon 8 deletion that also had impaired glucose tolerance [119]. The β cell ER was distended, islet BiP and CHOP expression were increased, and caspase 3 was activated. These models suggest that diabetes in Wolfram syndrome results from ER stress-induced β cell apoptosis. As for the Akita mice, *wfs1* knockout mice showed sex differences, with male animals having a worse phenotype [120]. In Wolfram syndrome patients, however, there are no clear gender differences. *Cisd2* knockout mice have also been generated to study Wolfram syndrome 2 [92]. Cisd2 deficiency triggers premature aging and nerve and muscle degeneration as a result of mitochondrial dysfunction and autophagic cell death. The *Cisd2* knockout mice develop optic nerve degeneration at 2–3 weeks of age. Glucose tolerance and insulin secretion of *Cisd2* knockout mice is mildly impaired, with no clear changes in β cell mass [92].

The NOD (non-obese diabetic) mouse is a model of autoimmune type 1 diabetes [121]. Tersey et al. showed that prior to the onset of diabetes in NOD mice, circulating proinsulin/insulin ratios increased and islet expression of BiP, sXBP1 and CHOP increased [122].

## 7 Novel disease-relevant human cell models

### 7.1 Human pluripotent stem cells

Human embryonic stem cells (hESC) and hiPSCs, collectively termed human pluripotent stem cells (hPSC) are unique cells due to their capability for unlimited self-renewal and differentiation into virtually any cell type. hESC lines are derived from the inner cell mass of pre-implantation embryos that are produced by *in vitro* fertilization. Thomson et al. generated the first hESC line in 1998 [123], and hundreds of hESC lines have been generated since (https://hpscreg.eu/). iPSCs are created by reprogramming terminally differentiated somatic cells back into pluripotency. Somatic cell reprogramming of human cells was described in 2007 by Takahashi et al. [124]. The first iPSC lines were generated by genome integrating retroviruses, which involves risks of insertional mutagenesis, unwanted residual expression of exogenous genes, which can hamper differentiation, or spontaneous reactivation of foreign genes during differentiation [125]. Today iPSCs are reprogrammed with integration-free methods, for instance using RNA based methods [126]. iPSCs have certain advantages over hESC; they allow generation of patient- and disease-specific hiPSCs, and they do not face the same ethical, political, and religious issues related to human embryo use. The bottleneck of disease modeling with hPSC is the identification of differentiation protocols to produce disease relevant cell types, such as pancreatic β cells. The current β cell differentiation methods involve several-week, multi-step protocols during which the cells are treated with growth factors and small molecules to guide β cell differentiation [127].

### 7.2 Modeling monogenic diabetes with hPSCs

iPSCs have been generated from a variety of type 1 and type 2 diabetic patient cells, and some of these have been differentiated into insulin-producing β cells [128], [129], [130], [131], [132].

iPSCs have also been derived from MODY patients. iPSCs were reprogrammed from fibroblasts from patients with *HNF1A* diabetes [133] and differentiated into insulin-positive cells. The differentiation was done with an embryoid body-mediated protocol, which produces low quantities of immature insulin-secreting cells; no comprehensive functional analyses were performed. iPSC lines have also been reprogrammed from MODY patients with mutations in *HNF4A*, *GCK*, *HNF1B*, and *CEL* [128]. These iPSCs will serve as highly valuable tools to investigate the role of these genes in pancreatic, islet and β cell development, and β cell function and survival. Teo et al. showed that mutant *HNF1B* iPSC-derived pancreatic progenitors have altered transcription factor networks with decreased PAX6 expression and retarded cell growth [134].

A similar approach was taken by Shang et al. to study the molecular mechanisms of β cell failure in Wolfram syndrome. Mutant *WFS1* iPSC-derived β cells displayed insulin secretion that was comparable to β cells derived from healthy individuals [79], but the cells had lower insulin content and increased signaling in all UPR branches (eIF2α phosphorylation, ATF4, BiP, sXBP1, and nuclear ATF6 expression). The synthetic ER stressors thapsigargin or tunicamycin impaired insulin secretion by WFS1 iPSC-derived β cells but did not affect healthy β cells. Interestingly, the UPR was reduced in WFS1 iPSC-derived β cells by treatment with the chemical chaperone 4-phenyl butyric acid (PBA).

### 7.3 Genome editing meets hPSCs in disease modeling

In 2012, the collaborative work of the Doudna and Charpentier laboratories [135] demonstrated that in the CRISPR (clustered regularly interspaced short palindromic repeats) system, a single protein, Cas9, is able to function as a designer of single-site specific nuclease by associating with an engineered single guide RNA (gRNA) [135]. This method allows easy and specific generation or correction of mutations in cells. CRISPR/Cas9 technology together with hPSCs thus provides a unique platform for the functional evaluation of mutations of interest.

This approach was used by McGrath et al. to study the role of neurogenin 3 in pancreas development. *NEUROG*^−/−^ hESCs generated pancreatic progenitor cells with equal efficiency compared to control hESCs, but their further differentiation into endocrine progenitors was obstructed [136]. The role of the transcription factors PDX1, RFX6, PTFA1, GLIS3, MNKX1, neurogenin 3, HES1 and ARX in pancreas development has been examined by CRISPR/Cas9 [137]. Bi-allelic inactivation of the first six genes causes pancreatic endocrine cell deficiency and neonatal diabetes [138], [139], [140], [141], [142], [143]. hiPSCs with a mutation in both GATA6 alleles have also been created using CRISPR/Cas9 [144]. These cells refused to differentiate into definitive endoderm, the first progenitor cell population for pancreatic cells. Using endodermal progenitor cell lines derived from hPSCs [145], differentiation was achieved of GATA6^−/−^ cells into dysfunctional β cells. The major differences between human and murine GATA6 loss-of-function [146] show the particular importance of the hPSC model.

### 7.4 Remaining challenges for hPSC disease modeling

Even better differentiation protocols need to be developed that mimic as closely as possible human embryogenesis in the cell culture dish and produce mature functional β cells. Advances in β cell differentiation in the past years have brought us closer to this goal. Endocrine cell differentiation from hESCs was described in 2005 by D'Amour et al. [147], showing differentiation with up to 80% efficiency into definitive endoderm using high Activin A (Nodal agonist). Soon after, improved protocols for β cell differentiation were published, but these produced immature, polyhormonal cells that resembled more fetal than adult β cells. In 2014, two groups independently reported sophisticated 7-stage differentiation protocols to generate insulin-secreting cells from hESCs [148], [149]. These cells expressed key markers of mature β cells, displayed glucose-stimulated insulin secretion *in vitro* and reversed diabetes upon transplantation in mice. Russ et al. studied the endocrine induction step for proper generation of β cells from iPSCs [150]. They reported that the precise generation of PDX1+ and then PDX1+/NKX6.1+ progenitors blocked precocious activation of NEUROG3 that could lead the development of polyhormonal cells, which further turn into α rather than β cells. The estrogen-related receptor γ activates mitochondrial function and is important for β cells to meet the high cellular energy demands needed for glucose responsiveness. Targeted expression of estrogen-related receptor γ in iPSC-derived β-cells facilitates their glucose-stimulated insulin secretion *in vitro* and *in vivo* [151]. Alternative methods for β cell differentiation have been created. Rather than exposing the cell to a complex cocktail of small molecules and growth factors, Saxena et al. created a lineage-control network combining vanillic acid-triggered expression switches for the transcription factors NEUROG3, PDX1, and MafA to program iPSCs into glucose-sensitive insulin-secreting β cells [152].

## 8 Therapeutic modulation of β cell ER stress and ER stress signaling

Based on the importance of ER stress and the UPR in β cell dysfunction and death, it seems a promising therapeutic target for diabetes (Table 1). One approach has been to use chemical or pharmaceutical chaperones, such as PBA and taurine-conjugated ursodeoxycholic acid derivative (TUDCA). These low molecular weight compounds stabilize protein conformation and improve ER folding capacity [153], [154]. Treatment of hepatocytes with TUDCA or PBA suppresses PERK/eIF2α signaling as well as JNK and XBP1 [155]. In leptin-deficient ob/ob mice with severe obesity and insulin resistance, PBA and TUDCA improved glucose homeostasis and decreased PERK and IRE1 phosphorylation and JNK activation [155]. In clonal rat β cells overexpressing human IAPP and exposed to thapsigargin or high glucose and palmitate, TUDCA and PBA decreased eIF2α phosphorylation and CHOP and ATF3 expression [156]. In man, PBA administration partially prevented insulin resistance and β cell dysfunction induced by intralipid infusion [157].

**Table 1:** Overview of therapeutic approaches to modulate β cell ER stress and ER stress signaling.

Therapeutic agent	Mechanism of action	Related references
Chemical chaperones (TUDCA, PBA)	Improve ER folding capacity	[153], [154], [155], [156], [157]
GSK2606414	Inhibit PERK	[158], [159], [160]
ISRIB	Prevent eIF2B inhibition by phospho-eIF2α	[160], [161], [162], [163]
Salubrinal	Inhibit eIF2α dephosphorylation	[164], [165], [166]
Guanabenz	Inhibit eIF2α dephosphorylation	[167], [168], [169]
Ca^2+^ stabilizers (Rycal S107, dantrolene)	Prevent ER Ca^2+^ leak	[83], [170], [172]
GLP-1 analogs (exenatide)	Induce ER chaperones and JunB Block SREBP1c and C/EBPβ	[175], [176], [177], [178], [181], [182]
Insulin	Relieve ER workload	[183]
Thiazolidinediones (pioglitazone)	Induce BiP and inhibit CHOP	[184]
Sulfonylurea	Facilitate SUR1 protein folding	[186]
PPAG	Resistance to lipotoxic ER stress	[187]
Azoramide	Improve ER folding capacity	[188]

Highly effective UPR inhibitors have been developed in recent years. GSK2606414 is a potent and selective PERK inhibitor that prevents translational attenuation [158]. GSK2606414 is neuroprotective in prion-infected mice but, not unexpectedly in light of the above-described impact of PERK loss-of-function, it induced hyperglycemia after 2–3 weeks of treatment [159] with extensive destruction of pancreatic exocrine tissue [160].

The ISR inhibitor ISRIB prevents the inhibition of eIF2B by phosphorylated eIF2α and thereby prevents translational inhibition and downstream signaling by ATF4 [161], [162], [163]. ISRIB improves memory in mice [161] and is neuroprotective in prion-infected mice without pancreas toxicity [160]. ISRIB partially restores protein synthesis (by 50–70%) compared to near total restoration by GSK2606414 (by 90–100%); this was suggested to explain the different impact on the exocrine pancreas [160]. The effects of ISRIB on β cell function and survival remain to be studied.

Agents that target eIF2α dephosphorylation directly have also been identified. Salubrinal was first identified as a selective inhibitor of eIF2α dephosphorylation that protects rat pheochromocytoma cells from ER stress-induced apoptosis [164]. In stark contrast to its salubrious effect in pheochromocytoma cells, salubrinal was toxic to pancreatic β cells and markedly potentiated free fatty acid-induced ER stress and apoptosis [165]. Salubrinal increased eIF2α phosphorylation to levels that are not tolerable by either rat or human β cells [165], [166]. Guanabenz is another chemical that prevents eIF2α dephosphorylation. It was suggested previously that guanabenz specifically disrupts GADD34-PP1 but not CReP-PP1 binding [167], although this mechanism of action very recently has been questioned [168]. By keeping eIF2α phosphorylation at a tolerable level, guanabenz was suggested to control translation such that it increases chaperone to substrate ratio and facilitates protein folding. The drug protects ER stressed HeLa cells and clonal rodent β cells that express Akita insulin [167]. In keeping with the effects of salubrinal, however, guanabenz sensitized β cells to fatty acid-induced ER stress and apoptosis [169].

Recent studies suggest that Ca^2+^ stabilizers allow to therapeutically modulate ER stress. The type 2 ryanodine receptor/Ca^2+^ release channel (RyR2) plays an important role in ER Ca^2+^ homeostasis. Patients with catecholaminergic polymorphic ventricular tachycardia due to RyR2 mutations were shown to have impaired insulin secretion and glucose tolerance, and a similar phenotype was observed in knockin mice harboring these human RyR2 mutations [170]. These leaky RyR2 channels cause mild islet ER stress and mitochondrial dysfunction. Pharmacologic stabilization of RyR2 by Rycal S107, which prevents stress-induced dissociation of the stabilizing subunit calstabin2 from RyR2 and prevents ER Ca^2+^ leak, improved insulin secretion and glucose tolerance in the knockin mice [170]. In WFS1-deficient β cells and neurons that also have altered ER Ca^2+^ homeostasis (see above), ER Ca^2+^ stabilization was also beneficial. The RyR blocker dantrolene, that suppresses ER-to-cytosol Ca^2+^ leakage [171], prevented β cell (and neuronal) apoptosis in WFS1 models by suppressing calpain activation [83]. Dantrolene was also shown to protect β cells from synthetic ERs stressors [172].

GLP-1 and glucose-dependent insulinotropic polypeptide (GIP) are incretin hormones that stimulate glucose-induced insulin secretion and β cell survival [173]. Exenatide is a long-lasting GLP-1 analog used to treat type 2 diabetes [174]. Several studies indicate that GLP-1 analogs are β cell protective by modulating the ER stress response. Exenatide and the cAMP inducer forskolin improve β cell survival induced by synthetic ER stressors [175] and saturated fatty acids [176]. Exenatide upregulates the ER chaperone BiP and the transcription factor JunB, thereby enhancing cellular defense mechanisms [176], [177]. The GLP-1 receptor agonist augments expression of the anti-apoptotic proteins BCL-2 and XIAP and prevents caspase 12 activation, thereby inhibiting mitochondrial apoptosis [176]. Exenatide also attenuates glucolipotoxic ER stress in β cells by blocking induction of the transcription factors SREBP1c and C/EBPβ [178]. In other cell types GLP-1 analogs further attenuate ER stress by improving SERCA2 expression and activity [179], [180]. *In vivo*, exenatide attenuates ER stress and β cell apoptosis in Akita mice and improves glucose levels [181]. A recent study showed that exenatide administration reduces glycemia in exon 8 deleted *wfs1*^−/−^ mice [182]. While β cell ER stress was not examined in this study, the data suggest that GLP-1 analogs hold promise for the treatment of Wolfram syndrome diabetes.

Early insulin treatment of heterozygous male Munich *Ins2* C95S mutant mice normalized glucose levels, reduced oxidative stress, and increased β cell mass [183], possibly by reducing demand for insulin production and thereby reducing ER stress.

Thiazolidinediones, such as pioglitazone, are peroxisome proliferator-activated receptor-γ agonists and insulin sensitizers used to treat type 2 diabetes. Maganti et al. reported an increase in β cell mass of pioglitazone-treated non-obese diabetic mice, possibly through induction of ATF4 and BiP and prevention of CHOP upregulation [184].

Sulfonylurea stimulate insulin secretion by binding to the regulatory subunit SUR1 of the β cell K_ATP_ channel. Inactivating mutations in *ABCC8*, encoding SUR1, lead to congenital hyperinsulinism. Folding mutant SUR1 is retained in the ER and targeted for ERAD [185]. Sulfonylurea have been shown to act as pharmacological chaperones and facilitate the folding, maturation, and ER-to-membrane trafficking of mutant SUR1 proteins [186], raising the intriguing suggestion that hyperinsulinism might be treated by these insulin secretagogues.

PPAG (phenylpropenoic acid glucoside) is a glucose-lowering compound of Rooibos (*Aspalathus linearis*). In high fat-fed mice PPAG increases β cell mass, probably by increasing β cell resistance to lipotoxic ER stress and apoptosis [187].

Azoramide was recently identified through ER functional screening assays that measure ER free chaperone content and protein folding capacity [188]. Azoramide protects against chemical, hypoxic, lipotoxic, and protein misfolding-induced β cell ER stress. It increases BiP and p58^IPK^ chaperone expression and ER folding capacity [188]. This makes azoramide a promising agent for the treatment of the ER stress-related diabetes.

## 9 Concluding remarks

Human islet and β cell transcriptomes, monogenic forms of diabetes, and pharmacological studies provide compelling evidence that unresolvable ER stress or dysregulation of eIF2α phosphorylation is ill tolerated by β cells and results in diabetes. The species-, disease-, and patient-relevant model of iPSC-derived β cells will further our understanding of the underlying pathogenic mechanisms. An increasing number of pharmacological approaches are available to modulate ER stress and the UPR. Some of these are in clinical use and others may find translation to the treatment of patients with monogenic or polygenic forms of diabetes.
